# The Cognitive Orientation to daily Occupational Performance approach in childhood‐onset disabilities

**DOI:** 10.1111/dmcn.16260

**Published:** 2025-03-03

**Authors:** Hortensia Gimeno, Helene Polatajko

**Affiliations:** ^1^ Barts Bone and Joint Health, Blizard Institute Queen Mary University of London London UK; ^2^ The Royal London Hospital and Tower Hamlets Community Children's Therapy Services Barts Health NHS Trust London UK; ^3^ Department of Occupational Science and Occupational Therapy, Rehabilitation Sciences Institute, Faculty of Medicine University of Toronto Toronto Canada

## Abstract

The Cognitive Orientation to daily Occupational Performance (CO‐OP) approach, a goal‐oriented intervention focused on participation, is designed to improve performance by addressing personal goals important to children and their families. Introduced in 2001, CO‐OP involves client‐chosen functional goals, identifying performance issues through a process of dynamic performance analysis, and guiding the discovery of cognitive strategies to enhance skill acquisition, all within a problem‐solving framework. The objectives of the approach are skill acquisition, strategy use, generalization, and transfer of learning. Developed within a research paradigm, a review of the literature indicates that CO‐OP research has expanded, documenting its use across various paediatric populations, including children with neurodevelopmental disorders, cerebral palsy, and movement disorders, addressing a myriad of functional goals. In this review we illustrate the iterative development of CO‐OP from single‐case experimental designs to randomized controlled trials to evaluate the approach. The Canadian Occupational Performance Measure and the Performance Quality Rating Scale are the most common outcome measures. Methodological advancements, limitations, and an initial exploration of mechanisms of action are discussed, providing a foundation for further research and clinical application. Recommendations include the use of consistent measures, robust longitudinal studies, implementation research, and health economic analyses.

## Graphical abstract

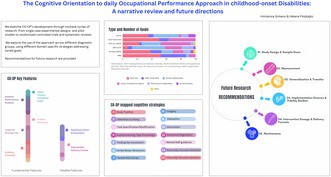

Plain language summary: https://onlinelibrary.wiley.com/doi/10.1111/dmcn.16314

Spanish translation of this Original Article is available in the online issue.

**Abbreviations**
CO‐OP: Cognitive Orientation to daily Occupational Performance
DCD: developmental coordination disorder
DPA: dynamic performance analysis
PQRS: Performance Quality Rating Scale
RCT: randomized controlled trial
SCED: single‐case experimental design

**What this paper adds**

- The Cognitive Orientation to daily Occupational Performance (CO‐OP) approach is now used across many diverse populations.
- Cognitive strategies are varied with 14 distinct types reported in the literature. Robust designs and implementation research in CO‐OP studies are needed.
- Recommendations for future research are provided including the need for definitive trials, consistent outcome measurement, and implementation studies.

The Cognitive Orientation to daily Occupational Performance (CO‐OP) approach^1^ was introduced in 2001 as an innovative intervention for children with developmental coordination disorder (DCD). The CO‐OP approach contrasted at the time of its development with traditional models by focusing directly on skill acquisition through cognitive strategy use, without specifically addressing underlying impairments. Over two decades, CO‐OP has expanded significantly, proving relevant for various populations, including children with cerebral palsy (CP), autism, and acquired brain injury.

In this narrative review we examine CO‐OP's development through multiple cycles of research, from single‐case experimental designs (SCEDs) and pilot studies, to randomized controlled trials (RCTs) and systematic reviews. We explore the use of the approach across different diagnostic groups, using different domain‐specific strategies addressing varied goals, noting its adaptability across diverse childhood disabilities. Recommendations for future research are provided.

## THE CO‐OP APPROACH

CO‐OP, first introduced in the literature in 2001^2^ as a novel intervention for children with DCD, is, first and foremost, a goal‐oriented approach designed to improve performance. In contrast to many rehabilitation approaches that seek to address underlying impairments and performance components, such as reducing tone or improving balance or fine motor abilities, goal‐oriented approaches focus explicitly on achieving specific functional goals.^3^ CO‐OP starts with the identification of goals; these then become the focus of intervention until achieved.^4^, ^5^, ^6^, ^7^ Other terms, such as task‐oriented and task‐specific training, are also used to describe interventions that focus on specific activity performance issues rather than underlying impairments and functional limitations. CO‐OP differs from other goal‐oriented or task‐oriented/specific interventions as it engages the patient/child as an active collaborator in the therapeutic process of problem‐solving to achieve their personal goals, starting with addressing the personal goals that children, young people, and their families identify as important to their everyday lives. CO‐OP is unique in that it uses a non‐directive approach to skill acquisition called guided discovery to enable problem‐solving, the core of the intervention, and has skill generalization and transfer as a specific aim.

CO‐OP is an individualized, client‐centred intervention underpinned by cognitive and motor learning theories. The CO‐OP approach was derived from the work of Meichenbaum and principles of cognitive behaviour therapy, where clients are taught a problem‐solving strategy and verbal self‐instruction to address unhelpful thinking patterns.^8^ In CO‐OP, the problem‐solving strategy has been adapted to address problems in the activity and participation domains. With its theoretical basis in learning and cognitive approaches, learning new skills is viewed as a problem‐solving exercise that should occur in the appropriate context (i.e., home or school) to facilitate appropriate skill acquisition, generalization, and transfer. CO‐OP is grounded in a range of theoretical perspectives, each of which contributes to the therapeutic approach.

The approach uses a framework consisting of seven key features, five of which are fundamental, to develop, apply, and generalize cognitive strategies to solve everyday performance problems.

The five fundamental elements of CO‐OP are as follows. (1) Client‐chosen functional goals: set by the child/young person, hence personally meaningful and relevant. (2) Dynamic performance analysis (DPA): involving closely observing and analysing how children and young people perform tasks, and identifying performance breakdowns and potential strategies that can be used. Ultimately, it is the children and young people who learn to carry out dynamic analysis of their own performance. This dynamic, iterative process is based on an analysis of the interaction between the individual, the task, and the environment. (3) Guided discovery: the process by which strategies to solve performance problems identified as part of DPA are solved. Guided discovery supports performance problem and strategy identification and, in turn, skill acquisition. Rather than providing direct instruction, the CO‐OP therapist facilitates clients to find their own solutions to their own problems, using questioning, coaching, working on one thing at a time, and being in synch with the client. (4) Cognitive strategy use: two types of cognitive strategies are employed—a global strategy and domain‐specific strategies. They support skill acquisition, generalization, and transfer. The global strategy supports metacognition by teaching a mnemonic ‘Goal‐Plan‐Do‐Check’, which is designed to support problem‐solving. It is used iteratively throughout the intervention. Goal—defines what the client wants to accomplish; Plan—develops a plan to achieve the goal; Do—executes the plan; and Check—evaluates the execution of the plan and its effectiveness and supports making adjustments/new plans, as necessary. By contrast, domain‐specific strategies, as the name implies, are specific to the performance breakdowns and are designed to overcome the breakdown; they are only used for a short time and are task, child, and environment‐specific/relevant. (5) Enabling principles: the theoretical underpinnings the therapist draws on to support skill acquisition, generalization, and transfer. In combination with DPA and guided discovery, four underlying enabling principles facilitate children's and young people's solving problems and achieving the goals they set. One of the most important principles is to promote generalization and transfer.

The approach includes two further features that are somewhat flexible in their delivery and implementation. (6) Significant other involvement: when working with children and young people, the involvement of parents and significant others is essential so that they become active supporters of the intervention itself. By learning how to implement DPA and guided discovery, parents and significant others are enabled to support the use of the intervention's key features in everyday life. This is important for generalization and transfer of learning beyond the therapy session. Significant others act as the bridge between the therapy intervention and the 24/7 real world of the child. (7) Intervention delivery format: the intervention format is divided into main phases. Phase 1 is the preparation phase and involves goal‐setting. Phase 2 is the acquisition phase, which generally comprises 10 intervention sessions. The first session involves teaching the global strategy Goal–Plan–Do–Check, and sessions 2 to 10 promote the child using the global strategy, learning how to do DPA, and discovering the use of domain‐specific strategies. Phase 3 is the closing re‐evaluation phase, the last session, where the goals are reviewed, and progress is evaluated.

The four key objectives of the CO‐OP intervention are (1) skill acquisition, (2) cognitive strategy use, (3) generalization, and (4) transfer of learning. CO‐OP actively engages the client in generating, testing, and refining their own strategies to achieve their own goals. This aspect of CO‐OP is intended to support transfer and generalization to other contexts and other goals, with benefits extending beyond the duration of the therapy intervention. The CO‐OP therapist's role is to help clients employ a problem‐solving strategy to enable them to develop their own effective strategies.

## CURRENT KNOWLEDGE ON THE USE OF THE APPROACH

Unlike many rehabilitation interventions, the CO‐OP approach originated in the research lab and started with proof‐of‐concept studies using SCEDs^9^ and case series^10^ to treat children with DCD. The approach, originally referred to as verbal self‐guidance, was renamed CO‐OP when analyses of intervention sessions revealed that a variety of cognitive strategies were driving the gains observed, not just verbal self‐guidance using a problem‐solving strategy.^11^ The early research was followed by replication and follow‐up studies. Finally, a pilot RCT^2^ was conducted. The evidence supporting the use of the approach for children with DCD spurred the investigation of the approach with other populations.^12^ A rapid search completed in March 2024 using the terms ‘CO‐OP’, ‘Cognitive Orientation to Occupational Performance’, and ‘Cognitive Orientation to daily Occupational Performance’ yielded 136 papers once duplicates were removed. Once papers were screened, 38 papers included children and young people, and had primary reported data. It shows that CO‐OP has expanded greatly since its introduction in 2001, not only in the number of studies available but also in several important ways. Many of the studies found in the literature are intervention studies evaluating its use with different populations, using a variety of research designs and approaches to measure outcomes, different delivery formats (individual vs. group delivery), and dosages, addressing a broad range of goals, with a variety of different types of strategies, and approaches to evaluating generalization and transfer.

CO‐OP has been used in several populations across the lifespan, from children aged 5 years old^13^, ^14^ to older adults experiencing stroke or mild cognitive delay.^15^ A systematic review of CO‐OP studies in stroke is available.^15^ Here we focus on the CO‐OP literature as it applies to children and young people.

The use of CO‐OP with children and young people with neurodevelopmental disorders has been recently synthesized in a systematic review.^16^ The approach has also been reported to have been used with children with other neurological conditions, such as CP^17^, ^18^, ^19^, ^20^, ^21^ and more severe movement disorders.^22^, ^23^, ^24^ It is the application of CO‐OP with people with more frank neurological disorders, first with patients with stroke^25^, ^26^, ^27^, ^28^, ^29^ and then with children and young people with hyperkinetic movement disorders, including dystonia^22^, ^23^, ^24^, ^30^ that challenged our initial premise and paradigms around motor skill acquisition. Table 1 shows different paediatric population groups investigated for applicability of the approach and the designs that were used.

**TABLE 1:** Summary of CO‐OP studies in children and young people (primary data).

Condition	Studies	Age ranges	Type of designs	Sample size	Dosage	Format
Acquired brain injury (including executive function)	*n* = 3	6–16 years	Pre‐post quasi‐experimental design (*n* = 1) SCED with replications (*n* = 2)	10 2 and 12	10–14 sessions	Individual
ADHD	*n* = 1	7–12 years	SCED with replications	6	12 sessions	Individual
Autism	*n* = 4	5–12 years	Case studies (*n* = 4)	7	10 sessions	Individual
DCD including DCD & ADHD	*n* = 17	5–16 years	SCED (*n* = 2) Pilot RCT (*n* = 2) RCTs (*n* = 2) Pre‐post‐group comparison (*n* = 6) Case studies (*n* = 5)	8 8 and 20 22 and 41 20, 12, 18, 8, 4 1–4	10 sessions (*n* = 10) 8–9 sessions (*n* = 2) 7 sessions of 2 hours (*n* = 1) 12 sessions (*n* = 1) Intense 2x50 minutes per day for 4 days (*n* = 1) 9.5 hours over 5 days plus group work (*n* = 1) 20 1 hour (*n* = 1)	Individual (*n* = 13) Group (*n* = 4)
Down syndrome	*n* = 2	7–19 years	SCED (*n* = 1) RCT (*n* = 1)	6 12	7–8 sessions 10 sessions	Individual
Cerebral palsy, spina bifida, and movement disorders	*n* = 11	4–28 years	SCED (*n* = 3) Pilot RCT (*n* = 1) RCT (*n* = 3) Multiple case study (*n* = 1) Crossover design (*n* = 1)	31 18 45, 32, 38 10 12	10 sessions weekly or twice a week 45–60 minutes	Individual (*n* = 10) Group (*n* = 1)
Other—learning difficulties	*n* = 1		Pre‐post quasi‐experimental	30	36 sessions	Individual

Abbreviations: ADHD, attention‐deficit/hyperactivity disorder; CO‐OP, Cognitive Orientation to daily Occupational Performance; DCD, developmental coordination disorder; RCT, randomized controlled trial; SCED, single‐case experimental design.

Typically, small studies have been run each time CO‐OP is tested in a new population to provide proof of concept. There is a particular strength in the use of SCED studies with multiple baselines and replications for many of these studies.^23^, ^24^, ^31^, ^32^ The use of SCED, particularly for rare disorders and other populations, is acknowledged to be preferable to small unpowered RCTs.^33^ SCED studies allow for an exploration of individual outcomes, as in a number of participants achieving their goals, furthering our understanding of potential best responders.

Most studies to date have explored the efficacy (or preliminary efficacy) of CO‐OP with different populations when delivered in a controlled research environment. The effectiveness of the intervention with CO‐OP delivered by clinicians (rather than in a research setting) has been explored in a small number of studies^17^, ^23^ and, in adults, an implementation science study across five inpatient rehabilitation units for people with stroke.^34^

### Design and measures

Table 1 shows primary data CO‐OP studies with study design and sample size. The majority of studies have delivered CO‐OP at an individual level and with five studies delivering CO‐OP in group format.^35^, ^36^, ^37^, ^38^, ^39^ When reviewing the 38 studies with primary data for CO‐OP studies, goals were measured subjectively in the majority of studies (*n =* 35, 92%) using the Canadian Occupational Performance Measure^40^ and in a lesser number of studies (*n* = 23, 61%) with the Performance Quality Rating Scale (PQRS) in its different forms.^41^, ^42^ The application of the PQRS with individuals with dystonia and consultations with children, young people, and families to understand and analyse the collected data (stakeholder consultation on the use of the PQRS as part of Gimeno's public involvement in research) revealed the need to ensure the PQRS was individualized and did not penalize their involuntary movements.^42^ Hence, we use the term PQRS‐individualized.

### Delivery formats and dosages

For individually delivered CO‐OP intervention studies, the majority of studies (*n* = 21, 55%) were conducted in 10 hourly sessions weekly.^2^, ^13^, ^14^, ^17^, ^23^, ^24^, ^43^, ^44^, ^45^, ^46^, ^47^, ^48^, ^49^, ^50^, ^51^, ^52^, ^53^, ^54^, ^55^, ^56^, ^57^ Three studies applied between 7 hours and 9 hours,^58^, ^59^, ^60^ four studies delivered 12 sessions,^20^, ^31^, ^61^, ^62^ and there were two studies with 14 sessions.^32^, ^63^ Dosage delivered in group formats was variable, with two studies reporting to deliver eight sessions,^37^, ^39^ 10 sessions in two other groups,^36^, ^38^ and one study delivering 20 hourly sessions.^35^

### Broad range of goals

A wide variety of client‐chosen goals have been addressed in different studies. Individual goals were reported in 23 studies and six studies categorized/themed goals in areas. Specific goals (*n* = 180) were reported in 29 of the primary studies identified across autism (*n* = 4, seven children and young people), Down syndrome (*n* = 2, 11 children and young people), DCD (including attention‐deficit/hyperactivity disorder) (*n* = 12, 74 children and young people), acquired brain injury and executive function (*n* = 3, 11 children and young people), CP and spina bifida (*n* = 6, 58 children and young people), and hyperkinetic movement disorders (*n* = 2, 19 children and young people). Broadly, goals are categorized across the studies reporting what goals children and young people worked on during CO‐OP sessions. A summary of the type of goals per population group is represented in Table 2. Goals described in these studies can be categorized across (1) self‐care (35% of goals); (2) daily living skills (12% of goals); (3) school‐related goals (23% of goals) with handwriting being one of the most frequent goals, reported 59 times; (4) leisure activities and sports (27% of goals); (5) planning and organization (8% of goals); and (6) social activities (2% of goals). Some differences are noted in the type of goals per population group. While leisure and sports activities were most frequently reported for children and young people with DCD (41% of goals), school‐related goals were more frequent in studies including autistic children (34%), and acquired brain injury and executive function (45%). Self‐care was the top goal theme identified by children and young people with hyplerkinetic movement disorders (53), CP and spina bifida (38%), Down syndrome (50%), and autism (34%).

**TABLE 2:** Type and number of goals identified in studies.

Condition and number of studies (number of children)	Types of goals/number of goals (*n*)						
	Self‐care goals	Daily living skills goals	School‐related goals	Leisure activities and sports goals	Planning and organization goals	Social activities goals	Other
Autism 4 (7)	10/29 (34%)	2/29 (7%)	10/29 (34%)	1/29 (3%)	3/29 (10%)	3/29 (10%)	0
Down syndrome 2 (11)	8/16 (50%)	2/16 (13%)	0	6/16 (37%)	0	0	0
DCD (including ADHD) 12 (74)	51/226 (23%)	6/226 (3%)	59/226 (26%)	92/226 (41%)	11/226 (5%)	0/226	4/226 (2%)
ABI and EF 3 (11)	2/31 (6%)	2/31 (6%)	14/31 (45%)	7/31 (23%)	5/31 (16%)	1/31 (3%)	0
CP and spina bifida 6 (58)	84/221 (38%)	33/221 (15%)	36/221 (16%)	31/221 (14%)	23/221 (10%)	8/221 (4%)	6/221 (3%)
HMD 2 (19)	30/57 (53%)	20/57 (35%)	2/57 (4%)	5/57 (9%)	0/57	0/57	0/57
Total 29 (180)	185/523 35%	65/523 12%	121/523 23%	142/523 27%	42/523 8%	12/523 2%	10/523 2%

Abbreviations: ABI, acquired brain injury; ADHD, attention‐deficit/hyperactivity disorder; CP, cerebral palsy; DCD, developmental coordination disorder, EF, executive function; HMD, hyperkinetic movement disorder.

## STRATEGY USE

Through the analysis of intervention sessions, the classes of domain‐specific strategies used by different populations have been identified in studies using the approach with children with DCD, Asperger syndrome, movement disorders, and people with stroke.^11^, ^31^, ^44^, ^52^, ^64^, ^65^ Domain‐specific strategies (key feature 4 described above) are not taught to the participants; rather, the participants discover such strategies when the therapists use guided discovery.

The first group of observed strategies was categorized from filmed intervention sessions with children with DCD and resulted in seven categories,^11^ summarized in Table 2 (strategy numbers 1–7). Since then, from work with other populations, new classes of domain‐specific strategies have emerged, such as relaxation and imagery seen in CO‐OP studies with clients with movement disorders and/or CP^31^ and stroke.^64^ They are included in Table 3 (strategy numbers 8–9). New domain‐specific strategies were analysed from 40 hours of video‐recorded CO‐OP intervention in children and young people with hyperkinetic movement disorders, including dystonia, with novel strategies identified.^31^ Those novel strategies observed during CO‐OP treatment sessions included distraction, emotion regulation, mental self‐guidance, and externally and internally focused attention, as described in Table 3 (strategies 10–14).

**TABLE 3:** CO‐OP known (mapped) cognitive strategies.

Strategy description	Description
**1. Body position**	Verbalization of attention to or shift of the body, whole or part, relative to the task
**2. Attention to doing**	Verbalization to cue attending to the doing of the task
**3. Task specification/modification**	Discussions regarding the specifics or modification of the task or parts of the task that facilitate motor performance
**4. Supplementing task knowledge**	Verbalization of the specifics of a task—things that cannot be discovered or that are not central to the focus of attention
**5. Feeling of movement**	Verbalization of attention to the feeling of a particular movement as it is being carried out
**6. Verbal motor mnemonic**	Client attaches a name to the task or a component of the task or body position, that evokes a mental image to guide motor performance
**7. Verbal rote script**	A rote pattern of words used to guide a sequence
**8. Imagery**	An active process during which a specific action is reproduced in the mind without any real movements
**9. Relaxation**	An attempt to return one's system to a state of tranquillity or to diminish effort
**10. Distraction (passive, active, increasing cognitive load)**	Something that draws the client's attention away from focusing on the task, or the bodily movements associated with the task (e.g. something that encourages a client to direct less attention to the task or components of the task)
**11. Emotion regulation**	A reminder to elicit behaviours or visualizations that reduce anxiety or tension, either before or during task performance
**12. Mental self‐guidance**	Any verbalization for the formation of mental images or likenesses of people/places/things that support task performance
**13. Externally focused attention**	Any verbalization of directing and sustaining attention to an object or location related to the task, outside the body (e.g. the final destination or the object being handled and carried)
**14. Internally focused attention**	Any verbalization of directing or sustaining attention to the body, whole or in part, that is actively being utilized during task performance

Abbreviation: CO‐OP, Cognitive Orientation to daily Occupational Performance.

### Approach to evaluating generalization and transfer

Generalizability beyond trained tasks and transfer has been reported in a recent scoping review^66^ containing studies included in the previous scoping review (with a different research question).^67^ Evidence of transfer was measured using four different indicators that included (1) transfer to untrained goals; (2) scores from other standardized assessments of activities, skills, or performance components; (3) scores from validated patient‐reported outcomes; and (4) anecdotal reports. Most studies addressed transfer (*n* = 25), with 16 evaluating transfer explicitly and nine addressing transfer with one of the indicators outlined above. The review's authors highlight the variability in approaches to measure transfer and the measures and definitions used, which limits comparison between studies.

## CRITIQUE OF EVIDENCE AVAILABLE

A major limitation in many of the available studies is the small sample size, with only over a third of the studies including more than 10 patients (median = 8.5, range 1–45). Typically, small studies are run each time CO‐OP is tested in a new population in an effort to provide proof of concept. The main group of children where CO‐OP has been validated and proof of concept (and beyond for some groups) established include DCD, autism, CP, acquired brain injury, executive function, and childhood‐onset hyperkinetic movement disorders, including rare genetic dystonia disorders. The literature shows that the research approach is predominantly the use of a SCED with replications. Even though SCED using multiple baselines has been reported in 10 CO‐OP studies, not all provided sufficient replications (minimum of N‐of‐1 with three replications)^13^, ^20^, ^23^, ^24^, ^31^, ^32^, ^45^, ^59^ as per guidelines.^68^ This has allowed for the development of this approach with populations, including rare disorders. However, more robust approaches should be given more attention to establish efficacy with a definitive trial (Recommendation 1).

A significant problem for the majority of studies has been the possibility of rater bias. Studies either used only one rater or failed to demonstrate (or report) interrater reliability. Blinding of the PQRS, although possible, is not always reported. Two studies clearly specified that blinding of PQRS was not achieved.^69^, ^70^ In the remaining studies, information is not provided to determine either whether blind assessment was used or how it was achieved^2^, ^14^, ^71^, ^72^ (Recommendation 2).

From our review of the literature, 13 (34%) of the studies used an extra goal untrained in therapy to measure transfer, showing it is a feasible and acceptable approach to children, young people, and their families. It is a relatively simple and less time‐consuming approach to measuring transfer, and its consistent use in clinical and research studies could support the pooling of data across studies (Recommendation 3).

Most studies to date have explored the efficacy (or preliminary efficacy) of the approach with different populations when delivered in a controlled research environment. While group comparison studies (pilot RCTs/RCTs) enable an understanding of mean change between groups, the SCED studies allow for an exploration of individual outcomes, as in a number of participants achieving their goals, furthering our understanding of potential best responders.

The effectiveness of the intervention with CO‐OP delivered by clinicians has not been explored in detail, and only a few studies report outcomes when the intervention is delivered by clinicians,^17^, ^23^ including an implementation science study across five inpatient rehabilitation units for people with stroke.^34^, ^73^ This is fundamental to understanding whether the approach can be delivered in clinical services with fidelity and whether it is acceptable to clinicians as well as children, young people, and families (Recommendation 4).

## FUTURE DIRECTIONS

### Recommendation 1. Study design and sample sizes

Large‐scale studies, including adequately powered RCTs, are required to validate CO‐OPs effectiveness across populations. When appropriate, adequately designed feasibility studies should proceed with definitive trials so that power sample calculations are based on big enough sample sizes.

Where RCTs are impractical, SCED studies should include sufficient replications to permit meta‐analyses, ensuring data consistency and rigour.

### Recommendation 2. Consistency of measurement for meta‐analysis

The Canadian Occupational Performance Measure and the PQRS are two measures recommended in the CO‐OP approach to evaluate the outcome of the intervention. Consistent use and reporting of these two measures across studies would facilitate future pulling of the data to report effects in meta‐analysis studies for skill acquisition and transfer. Blinding of the PQRS is possible and encouraged.

### Recommendation 3. Generalization and transfer

Current evidence supports CO‐OP's ability to facilitate skill acquisition and immediate application. Transfer was not measured in all studies, but it has been shown to be one of the key elements differentiating CO‐OP from other interventions. We suggest the use of a fourth and possibly fifth goal to measure the effect of transfer of the intervention to untrained goals. The use of health economic analysis to capture the intervention's benefit beyond trained goals could address a significant gap in knowledge to support clinical services and commissioning.

Longitudinal studies are also needed to examine the sustainability of these skills over time and their transferability to untrained tasks and new environments. Evaluating long‐term outcomes will help to understand the maintenance of intervention improvement and whether further sessions are guaranteed.

### Recommendation 4a. Implementation science and fidelity studies

The literature is lacking in information on training, fidelity, and implementation research. As CO‐OP is disseminated to broader clinical and educational settings, research on training methods for therapists and educators is crucial. Implementation science studies can identify best practices for training, support mechanisms for ongoing professional development, and strategies for overcoming barriers to implementation in various contexts. Training and implementation studies are necessary for ensuring CO‐OP's scalability, exploring best practices for clinician training, and assessing fidelity in real‐world settings.

### Recommendation 4b. Intervention dosage and delivery formats

The optimal dosage and formats for CO‐OP interventions have not been systematically explored. While the standard approach involves 10 sessions, variations in frequency, duration, and group versus individual formats should be explored to determine the most effective and efficient models. Understanding these variables can lead to more tailored and accessible intervention plans, potentially reducing the burden on families and health care systems.

### Recommendation 5. Underlying mechanisms of change

Studies that identify active ingredients and mechanisms of change, including outcomes around transfer as defined above, are lacking. In order to understand the mechanism of action for CO‐OP, active processes and/or components of the treatment have to be identified and how they result in positive outcomes tested. Understanding why CO‐OP works, through which processes, and how change comes about across different populations would enable us to understand the mechanisms of therapeutic change. Understanding whether there are specific mediators and moderators of efficacy will help us understand the underlying mechanisms of action. Neurosciences evidence, such as understanding the pathway of motor activity and where CO‐OP acts in terms of neuronal pathways and interactions, may be helpful to refine the intervention and provide personalized treatment. Understanding CO‐OP's impact on neurodevelopment and cognitive‐motor pathways, especially in children with complex disabilities, could refine the approach and guide personalized treatment.

## CONCLUSION

This narrative review indicates that the CO‐OP literature has expanded in a number of important ways since its introduction in 2001. Taken as a whole, the literature indicates that CO‐OP can effectively support skill acquisition, generalization, and transfer for a number of populations, both child and adult, across a broad range of goals using various strategies. The literature could be improved by using more robust research designs and the addition of research on training, fidelity, and implementation.

## Data Availability

Not applicable.
